# Supplementary material for: A cross-scale framework for evaluating flexibility values of battery and fuel cell electric vehicles
Source: Nat Commun. 2024 Jan 4;15:280. doi: 10.1038/s41467-023-43884-x (PMC10766983; doi:10.1038/s41467-023-43884-x)
Supplement: Supplementary file 1 — Supplementary information [file 41467_2023_43884_MOESM1_ESM.pdf]

Supplementary Information for

A Cross-Scale Framework for Evaluating Flexibility Values of Battery and Fuel Cell Electric Vehicles

Ruixue Liu[1*], Guannan He[2,3,4,5*†], Xizhe Wang[1], Dharik Mallapragada[6], Hongbo Zhao[7], Yang Shao-Horn[6,8,9,10†], Benben Jiang[1†]

* These authors contributed equally to this work
† Corresponding authors: gnhe@pku.edu.cn, shaohorn@mit.edu, bbjiang@tsinghua.edu.cn.

Affiliations

1 Department of Automation, Beijing National Research Center for Information Science and Technology, Tsinghua University, Beijing, China

2 Department of Industrial Engineering and Management, College of Engineering, Peking University, Beijing, China

3 National Engineering Laboratory for Big Data Analysis and Applications, Peking University, Beijing, China

4 Institute of Carbon Neutrality, Peking University, Beijing, China

5 Peking University Changsha Institute for Computing and Digital Economy, Beijing, China

6 MIT Energy Initiative, Massachusetts Institute of Technology, 77 Massachusetts Avenue, Cambridge, MA, USA

7 Department of Chemical and Biological Engineering, Princeton University, Princeton, NJ, USA

8 Department of Mechanical Engineering, Massachusetts Institute of Technology, 77 Massachusetts Avenue, Cambridge, MA, USA

9 Research Lab of Electronics, Massachusetts Institute of Technology, 77 Massachusetts Avenue, Cambridge, MA, USA

10 Department of Materials Science and Engineering, Massachusetts Institute of Technology, 77 Massachusetts Avenue, Cambridge, MA, USA

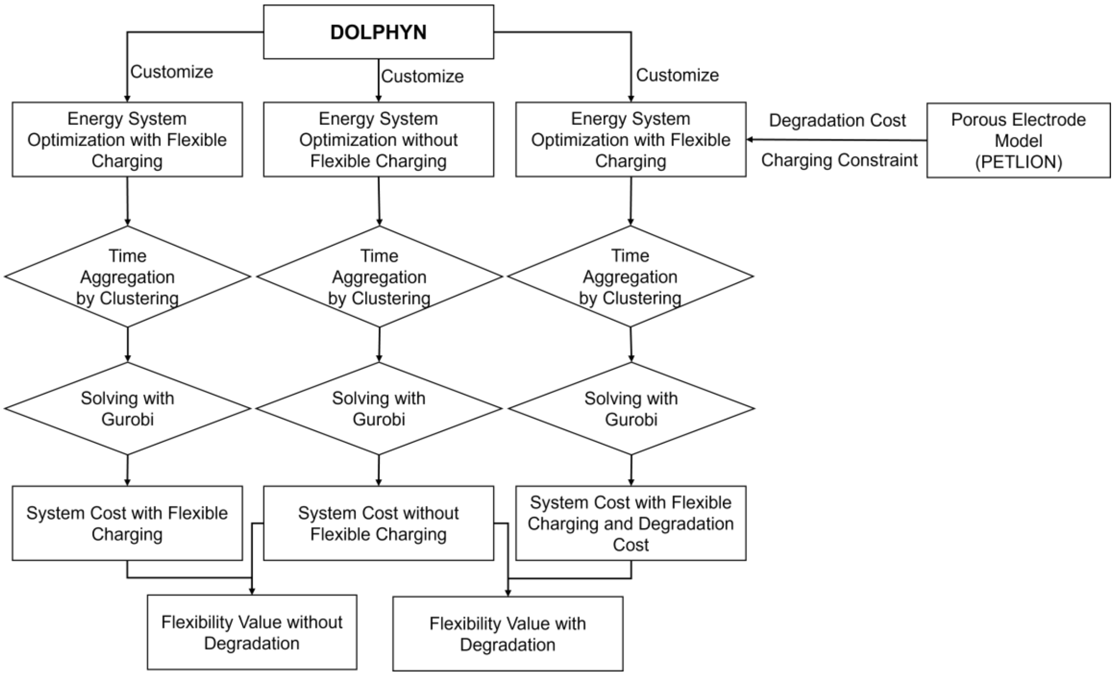

Supplementary Figure 1. The flow diagram for flexibility value computation of FCEVs and BEVs with battery degradation considered. The DOLPHYN model is customized for energy systems with different flexible charging settings, i.e. energy system with flexible charging, without flexible charging, and with flexible charging and battery degradation cost considered. The degradation cost is computed by Eq.(8) in the "Methods-Degradation cost calculation" Section with the cycle life L_{bat} obtained by a micro-level porous electrode theory-based model. After selecting several representative weeks from 7-year data of renewable generation and electricity demand using clustering techniques, the optimization problems under operational constraints are solved by Gurobi with battier methods. Then the least system costs of energy systems with and without flexible charging, and with flexible charging and degradation cost are obtained (Eq. 1-4 in the "Methods-Flexibility value calculation" Section). Finally, the flexibility values are figured out by computing the difference values between corresponding optimized least system costs (Eq. 5-7 in the "Methods-Flexibility value calculation" Section).

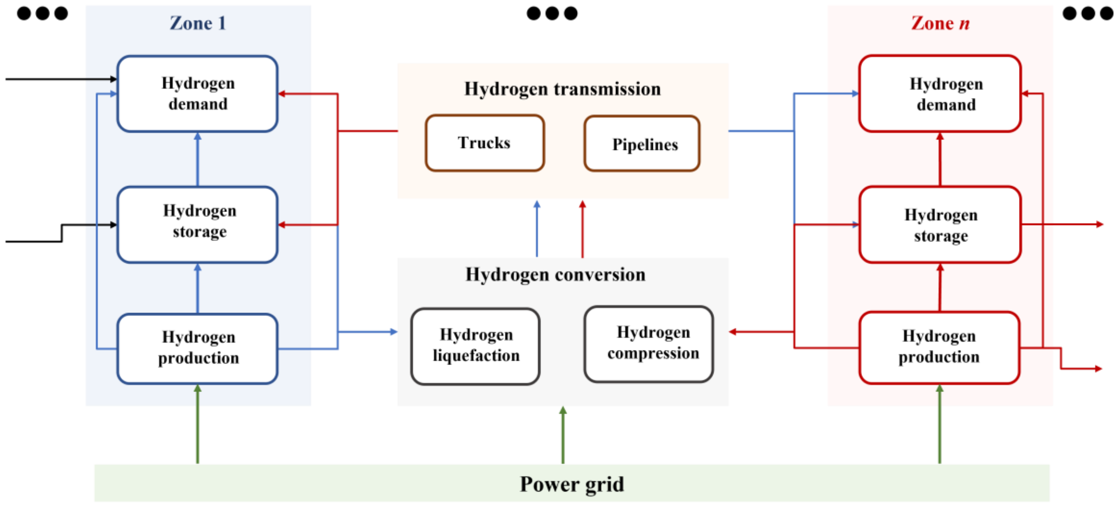

Supplementary Figure 2. The schematic of the hydrogen supply chain involved in the DOLPHYN model.

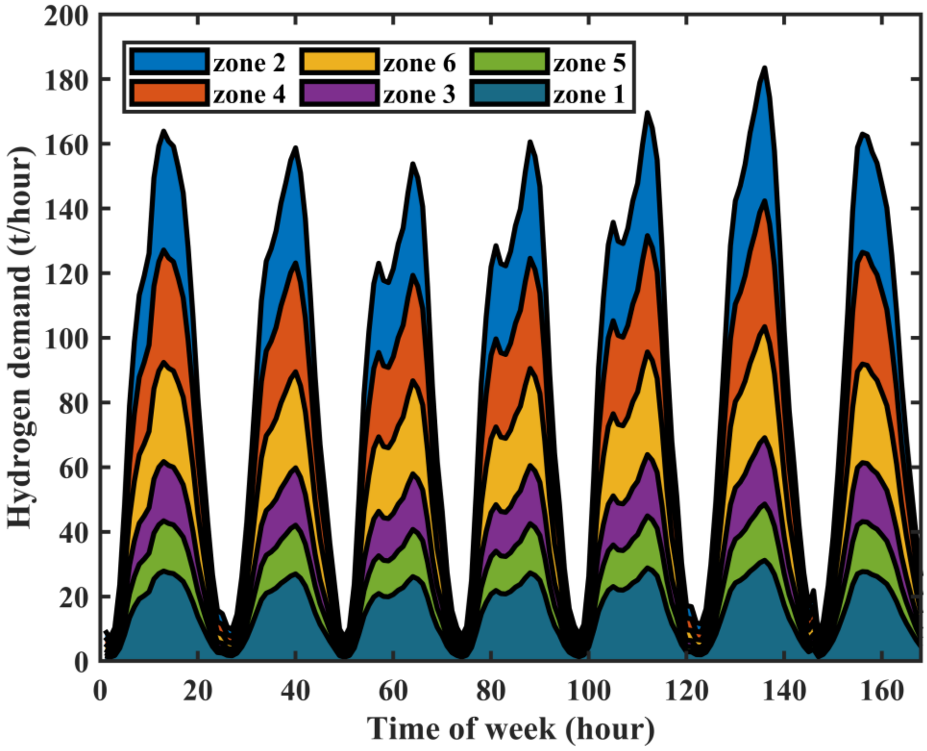

Supplementary Figure 3. Hydrogen demand at different zones. Hourly H_2 demands profiles for each zone. Corresponding zones are shown in Ref. 1.

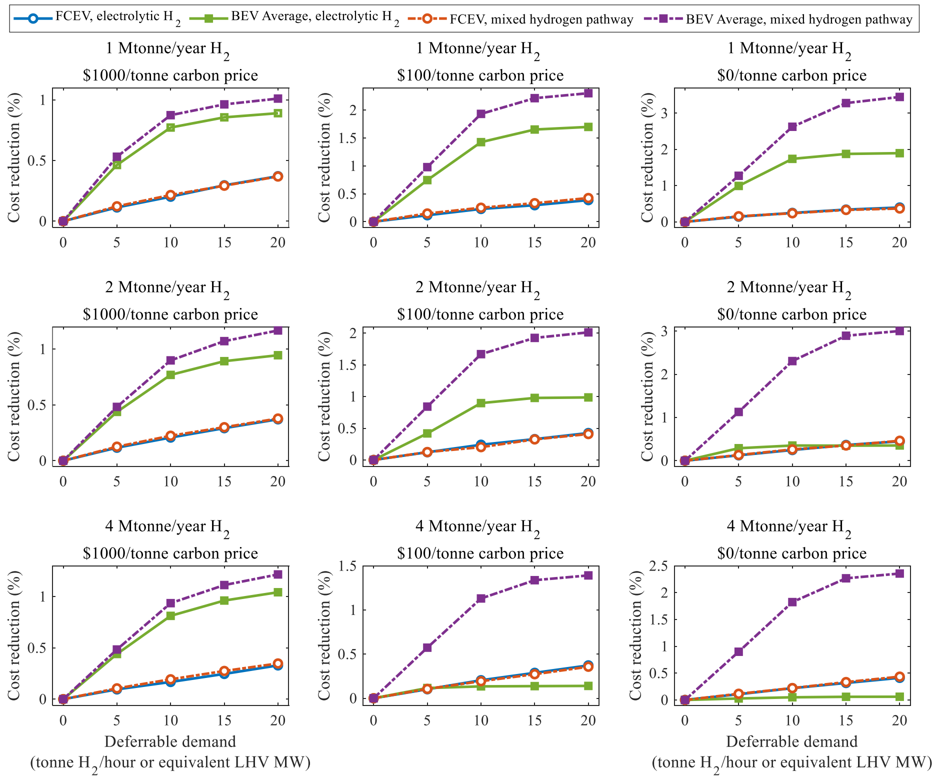

Supplementary Figure 4. The net values of flexible BEV and FCEV charging with a charging window of 1 h under different hydrogen pathways. The lines with circle markers denote the flexibility values of FCEVs, while the lines with solid square markers denote those of BEVs. The solid lines represent the electrolytic hydrogen only pathway, and the dashed-dotted lines are for the mixed hydrogen pathway incorporating both natural gas with carbon capture and storage (NG with CCS) and electrolytic generation.

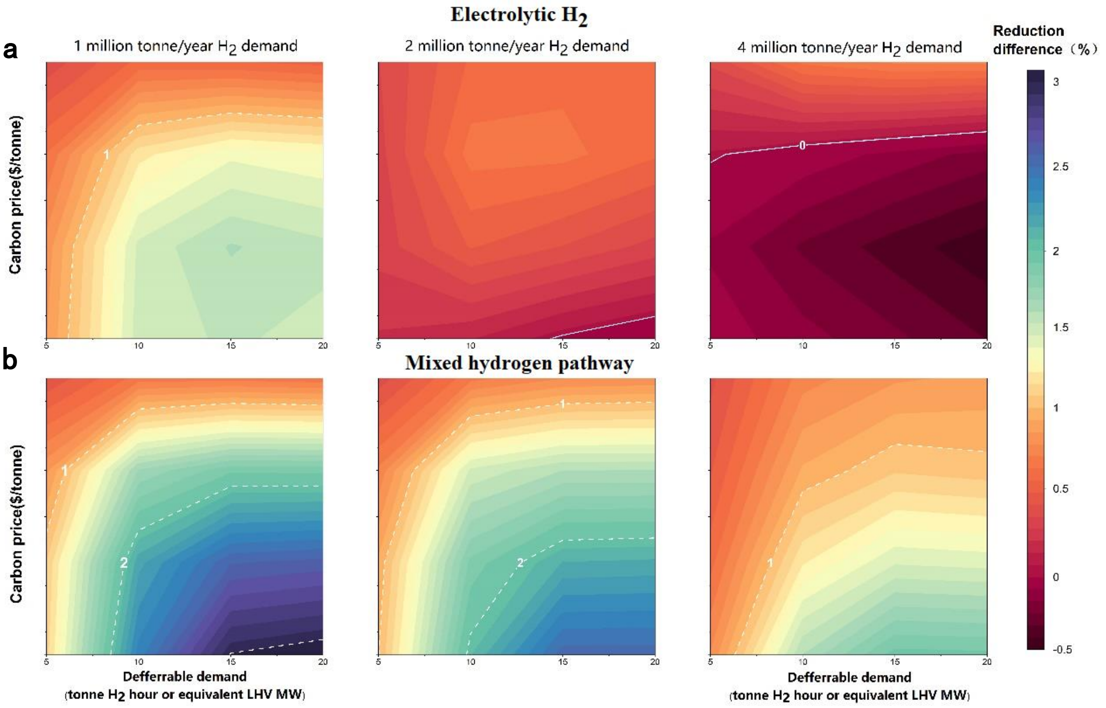

Supplementary Figure 5. The net value (system cost reduction) difference between flexible BEV and FCEV charging with a charging window of 1 h in different hydrogen generation pathways. **a**. Using electrolysis only for hydrogen production (namely, "Electrolytic H_2"), **b**. Using both electrolysis and NG with CCS, namely, "Mixed hydrogen pathway". Panels from left to right are for the H2 demand of 1 Mtonne/year, 2 Mtonne/year, and 4 Mtonne/year. The vertical coordinates are for different carbon prices. And the colors from red to blue denote gap magnitudes of flexibility values of BEVs and FCEVs.

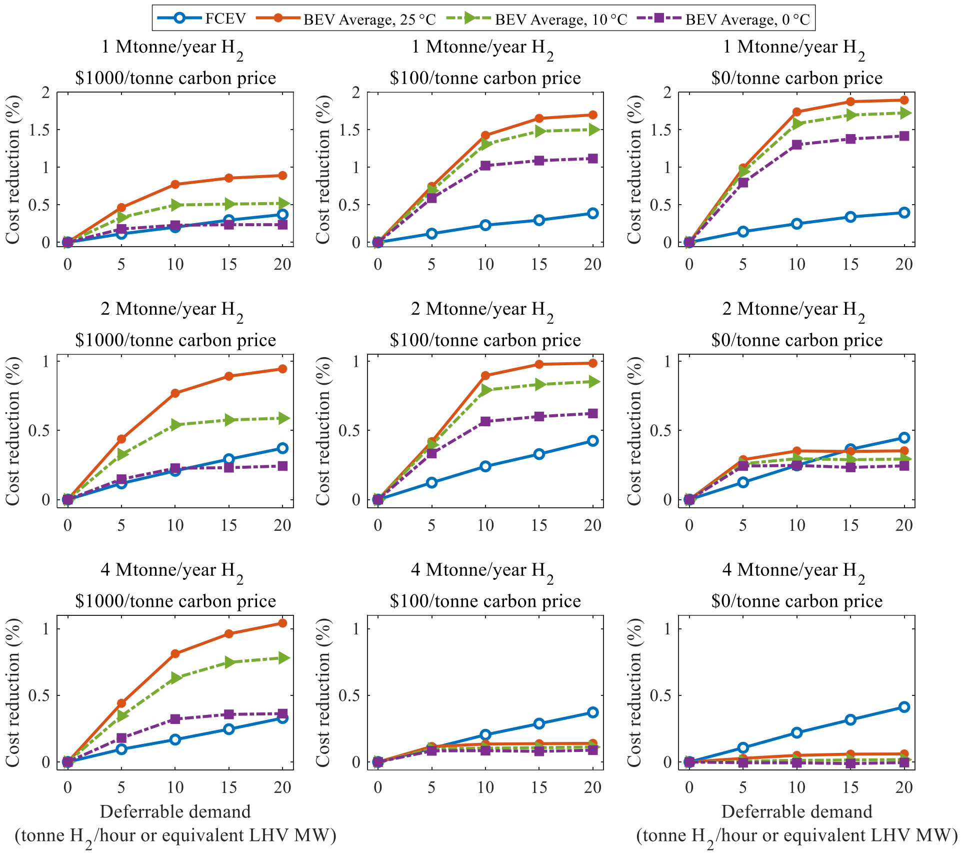

Supplementary Figure 6. The net values of flexible BEV and FCEV charging under a range of service temperatures. The blue lines with circle markers denote the cost reduction due to the flexibility of FCEVs. The lines in orange, green, and purple indicate the flexibility values of the BEV average charged within 1 h at 25℃ (marked as "BEV Average, 25℃"), at 10℃ (marked as "BEV Average, 10℃"), and at 0℃ (marked as "BEV Average, 0℃"), respectively.

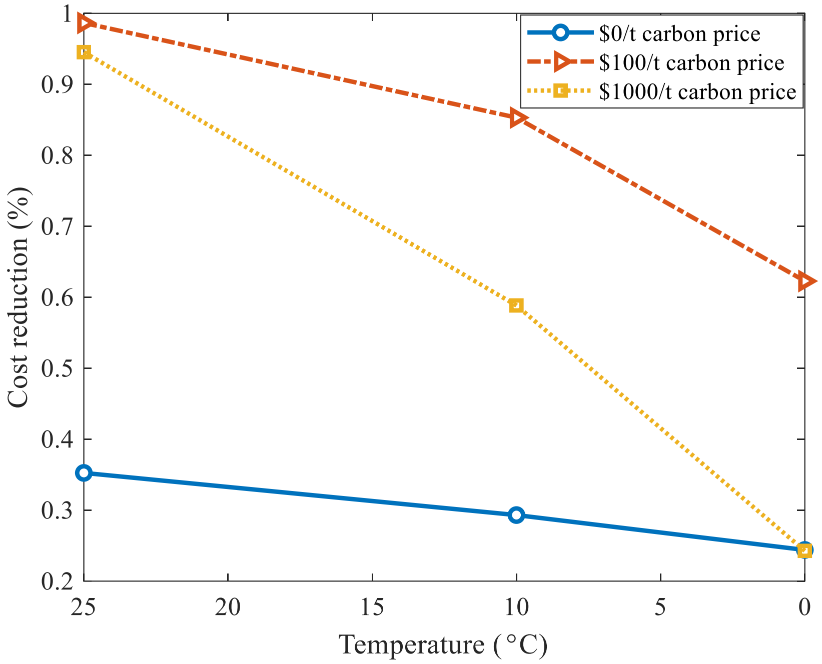

Supplementary Figure 7. The cost reduction of the BEV Average case with 20% deferrable demand and 2 million tonne/year H_2 demand at different temperatures. The blue, orange, and yellow lines are for carbon prices of $0, $100, and $1000 per tonne, respectively. A nonlinear relationship between the cost reduction and temperature of BEVs is observed, with a steeper slope at a colder temperature.

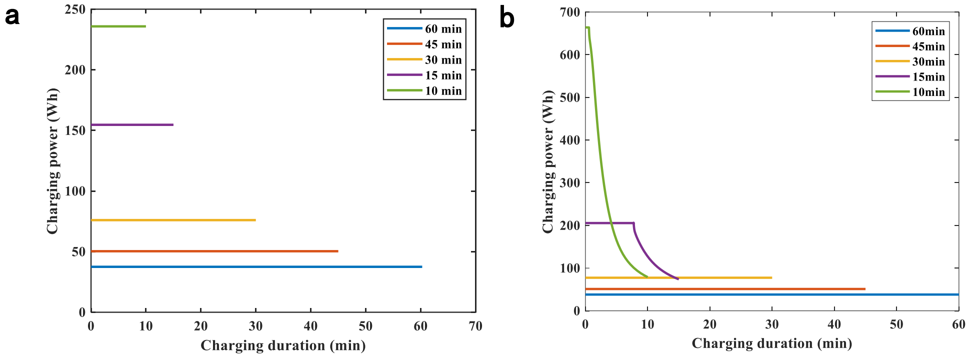

Supplementary Figure 8. Charging protocols for the five charging-time demands of 60 min, 45 min, 30 min, 15 min, and 10 min. **a**. Charging protocols at 25 °C and 10 °C. **b**. Charging protocols at 0 °C. At 0 °C, larger constant power is required at fast charging conditions. Then, constant voltage (CV) charging exists in these fast-charging protocols, such as 15 min or 10 min. The proportion of constant power (CP) charging duration decreases as the total charging time becomes shorter.

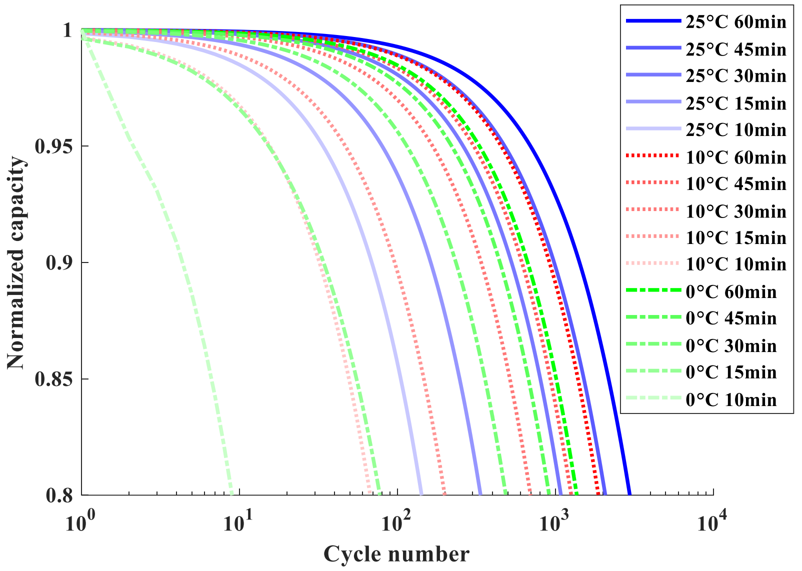

Supplementary Figure 9. Battery degradation trajectories as battery cycling, using different charging protocols at various service temperatures. Blue-solid, red-dotted, and green-dashed-dotted lines denote 25 °C, 10 °C, and 0 °C, respectively. Gradually changing colors from deep to shallow represent the corresponding charging duration time from long to short. The battery cycle life defined here is the cycle number corresponding to a reduction in the cell capacity to 80% of the nominal capacity.

Supplementary Table 1. Parameters for generation and storage technologies in the power sector for the year 2045[2,3]. CAPEX: capital cost; FOM: fixed operational and maintenance cost; VOM: variable operational and maintenance cost; PV: photovoltaic; CCGT: combined cycle gas turbine; OCGT: open cycle gas turbine; CCGT w/CCS: combined cycle gas turbine with carbon capture and storage.

Technology	Onshore Wind	Offshore Wind	Utility PV	Distributed PV	Li-ion Battery	Pumped Hydro	CCGT	OCGT	CCGT w/CCS	Nuclear
Power CAPEX (10^3\$/MW)	1074	2179	725	882	119	1966	936	854	2080	6048
Energy CAPEX (10^3\$/MWh)	--	--	--	--	136	--	--	--	--	--
FOM (10^3\$/MW-year)	35	59	8	6	2	44	13	11	27	119
VOM (\$/MWh)	--	--	--	--	3	--	2	4	6	2
Heat Rate (MMBTU/ MWh)	--	--	--	--	--	--	6	10	8	10
Round-trip Efficiency	--	--	--	--	85%	80%	--	--	--	--
Lifetime (years)	30	30	30	30	15	50	30	30	30	30

Supplementary Table 2. Parameters for H_2 generation and gas-to-power (G2P) technologies. CAPEX: capital cost; SMR: steam methane reformer; SMR w/CCS: steam methane reformer with carbon capture and storage (CCS); CCGT: combined cycle gas turbine.

	Electrolysis[4]	SMR[4]	SMR w/CCS[4]	Fuel Cell[5]	CCGT-H_2[2]
Unit CAPEX	300-700 $\$/kW_e$	910 $\$/kW_{H_2}$	1280 $\$/kW_{H_2}$	1264 $\$/kW_e$	1171 $\$/kW_e$
Lifetime (years)	10	25	25	10	25
Efficiency (LHV)	74%	76%	69%	60%	65%
Emission Intensity (tonne CO_2/tonne H_2)	0	8.9	1.0	0	0

Supplementary Table 3. Parameters for H_2 transmission and storage technologies. CAPEX: capital cost; OPEX: operational cost; A: cost and electricity consumption proportional to pipeline length; B: cost and electricity consumption irrelevant to pipeline length; C: truck and tank storage compression related costs and electricity consumption.

	Pipeline	Gas Tank	Liquid Truck	Gas Truck
Unit capacity	38.8 tonne/hour[4]	0.3 tonne[6]	4 tonne[6]	0.3 tonne[6]
CAPEX	3.72 M$/mile[4,7]	0.58 M$/tonne[6]	0.2 M$/tonne[6]	1 M$/tonne[6]
Compression CAPEX (A) ($/mile-unit)	700[8,9]	0	0	0
Compression CAPEX (B) (M$/unit)	0.75	0	0	0
Compression Electricity (A) (MWh/tonne-mile)	0.014	0	0	0
Compression Electricity (B) (MWh/tonne)	1	0	0	0
Unit OPEX ($/mile)	0	0	1.5	1.5
Compression CAPEX (C) ($/(tonne/hr))	0	0.5[6]	32[6]	1.5[6]
Compression Electricity (C) (MWh/tonne)	0	2[8,9]	11[8,9]	1[8,9]
Boil-off Rate	0	0	3%	0
Lifetime (years)	40	12	12	12

Supplementary Table 4. Additional parameters of the DOLPHYN model.

Discount Rate	5.4%
Power Transmission Expansion Cost	1600/MW-mile
Power Transmission Loss	1%/100 miles
Value of Lost Load (Electricity)	$20,000/MWh
Value of Lost Load (Hydrogen)	$1,000/kg
Gas Price	$5.4/MMBTU
CO_2 Transportation and Storage Cost	$20/tonne

Supplementary Table 5. Battery parameters for BEVs (the Base column) and the sensitivity to electrode thickness and porosity [10,11].

Cell Parameters	Base	-10% Cathode thickness	+10% Cathode thickness	-10% Anode thickness	+10% Anode thickness	+10% Cathode porosity	-10% Cathode porosity	+10% Anode porosity	-10% Anode porosity	-20% Cathode thickness	+20% Cathode thickness
Electrode Length (m)	0.3	0.3	0.3	0.3	0.3	0.3	0.3	0.3	0.3	0.3	0.3
Electrode Width (m)	0.1	0.1	0.1	0.1	0.1	0.1	0.1	0.1	0.1	0.1	0.1
Layers Per cell (m)	20	20	20	20	20	20	20	20	20	20	20
Voltage	4.2	4.2	4.2	4.2	4.2	4.2	4.2	4.2	4.2	4.2	4.2
**Anode**	**LiC6**										
Thickness (m)	4.8e-5	4.8e-5	4.8e-5	4.32e-5	5.28e-5	4.8e-5	4.8e-5	4.8e-5	4.8e-5	4.8e-5	4.8e-5
Porosity	0.3	0.3	0.3	0.3	0.3	0.3	0.3	0.33	0.27	0.3	0.3
Volume (m^3)	2.88e-5	2.88e-5	2.88e-5	2.592e-5	3.168e-5	2.88e-5	2.88e-5	2.88e-5	2.88e-5	2.88e-5	2.88e-5
Density (kg/m^3)	2260	2260	2260	2260	2260	2260	2260	2260	2260	2260	2260
Active Mass (kg)	1.95e-2	1.95e-2	1.95e-2	1.76e-2	2.15e-2	1.95e-2	1.95e-2	2.15e-2	1.76e-2	1.95e-2	1.95e-2
Energy Density (mAh/g)	330	330	330	330	330	330	330	330	330	330	330
Price ($/kg)	15	15	15	15	15	15	15	15	15	15	15
Capacity (Ah)	6.4437	6.4437	6.4437	5.7993	7.0881	6.4437	6.4437	7.0881	5.7993	6.4437	6.4437
Cost ($)	0.2929	0.2929	0.2929	0.2636	0.3222	0.2929	0.2929	0.3222	0.2636	0.2929	0.2929
**Cathode**	**NMC**										
Thickness (m)	4.16e-5	3.744e-5	4.576e-5	4.16e-5	4.16e-5	4.16e-5	4.16e-5	4.16e-5	4.16e-5	3.328e-5	4.992e-5
Porosity	0.3	0.3	0.3	0.3	0.3	0.3	0.3	0.33	0.27	0.3	0.3
Volume (m^3)	2.50e-5	2.246e-5	2.746e-5	2.50e-5	2.50e-5	2.50e-5	2.50e-5	2.50e-5	2.50e-5	1.997e-5	2.995e-5
Density (kg/m^3)	4670	4670	4670	4670	4670	4670	4670	4670	4670	4670	4670
Active Mass (kg)	3.50e-2	3.15e-2	3.85e-2	3.50e-2	3.50e-2	3.85e-2	3.15e-2	3.50e-2	3.50e-2	2.80e-2	4.20e-2
Energy Density (mAh/g)	170	170	170	170	170	170	170	170	170	170	170
Price ($/kg)	20	20	20	20	20	20	20	20	20	20	20
Capacity (Ah)	5.9447	5.3503	6.5392	5.9447	5.9447	6.5392	5.3505	5.9447	5.9447	4.7558	7.1337
Cost ($)	0.6994	0.6294	0.7693	0.6994	0.6994	0.7693	0.6294	0.6994	0.6994	0.5595	0.8393
Cell Capacity (Wh)	24.97	22.47	27.06	24.36	24.97	27.06	22.47	24.97	24.36	19.97	27.06
Cell Cost ($)	0.99	0.92	1.06	0.96	1.02	1.06	0.92	1.02	0.96	0.85	1.13
Number of cell per kWh	40.05	44.50	36.95	41.06	40.05	36.95	44.50	40.05	41.06	50.06	36.95
Additional cost per kWh	0.00	1.30	-0.49	-0.21	1.17	-0.49	1.30	1.17	-0.21	2.93	2.09
Cost per kWh	200.00	201.30	199.51	199.79	201.17	199.51	201.30	201.17	199.79	202.93	202.09

Supplementary Table 6. Battery cycle life obtained by the PET-based model with various charging-time protocols and temperatures. "Average" denotes the mean cycle life of the simulated values obtained by four charging-time protocols (60 min, 45 min, 30 min, 15 min), corresponding to the "Average" case in the manuscript. "Extreme" indicates that all batteries for the BEV fleet are charged in 10 min.

Temperature	60 min	45 min	30 min	15 min	**Average**	10 min (**Extreme**)
25℃	2962	2074	1086	337	1615	143
10℃	1875	1262	697	200	1009	68
0℃	1368	915	487	78	712	9

Supplementary Note 1. Charging protocol design

Batteries are cycled during the 30%–80% state of charge to avoid overcharging or overdischarging, with various charging protocols but identical constant current discharging protocols. The constant power constant voltage (CP-CV) charging strategy is applied. CV charging is used after CP charging hitting the upper cutoff voltage. In our simulation, CV charging only occurs in situations with a lower temperature of 0 °C and a shorter charging duration of 10-min or 15-min (Supplementary Fig. 5b). Various charging protocols are defined by different charging durations of 60 min, 45 min, 30 min, 15 min, and 10 min. The charging protocols at different temperatures are shown in Supplementary Fig. 5. All the batteries are discharged by the constant current (CC) protocol at a 1C rate.

Supplementary Note 2. Battery degradation

The capacity degradation trajectories of batteries for BEVs under various charging protocols and service temperatures are displayed in Supplementary Fig. 6. The differences between battery degradation trajectories at 25°C (solid lines) illustrate that the battery cycle life shortens as decreasing charging time. Large gaps between gradually lighter blue lines indicate that when charging time is less than 30 min, the degradation of battery is considerably faster than that of longer charging times, with a notably reduced cycle life observed. Similar phenomena can also be observed from these trajectories at 10°C (dotted lines) and at 0°C (dashed-dotted lines). At 25°C, the battery cycle life with 15 min charging reduces by 88.6% compared with that with 60 min charging, while the reduction ratio reaches 95.2% for 10 min charging. The battery lifetime decreases by more than 60% when charging time becomes half shorter. As battery cycling, solid electrolyte interface (SEI) grows at the anode, which decreases the anode porosity and increases the overpotential and the transport resistance of Li-ions[12]. The increasing impedance reduces capacity due to increased voltage loss during discharge. When the overpotential is significantly large, lithium plating at the anode is preferred compared to intercalation into the graphite. Lithium plating further decreases the porosity and accelerates the impedance build-up and capacity degradation.

Using the same charging protocol, the battery lifetimes decrease by over 30% and 50% at 10°C and 0°C, respectively, compared with those at 25°C. For the five charging protocols from long to short duration, the cycle life reduction ratios at 10°C are 36.7%, 39.2%, 35.8%, 40.6%, and 52.4%, in contrast to the cycle lives at 25°C. Compared with those at 10°C, the corresponding reduction ratios at 0°C are 27.0%, 27.5%, 30.1%, 61.0%, and 86.8%. The results show that batteries degrade nonlinearly as temperature decreases. Especially under 10 min charging, the lifetime at 0°C decreases up to a

93.7% compared with that at 25°C and with 60 min charging. The reason is that a lower temperature could facilitate porosity shrinkage and slow down the lithium-ion transport, which worsens lithium plating and thereby impedes lithium-ion intercalation.

Supplementary References

1. He, G., Mallapragada, D. S., Bose, A., Heuberger, C. F. & Gençer, E. Hydrogen supply chain planning with flexible transmission and storage scheduling. *IEEE Trans. Sustain. Energy* 12, 1730–1740 (2021).
2. National Renewable Energy Laboratory, 2021 Annual Technology Baseline (ATB), 2021, https://atb.nrel.gov/.
3. U.S. Energy Information Administration, Annual Energy Outlook 2018, With Projections to 2050, 2018.
4. The Future of Hydrogen, IEA technical report, 2019.
5. Manufacturing Cost Analysis of 100 and 250 kW Fuel Cell Systems for Primary Power and Combined Heat and Power Applications, Battelle Memorial Institute technical report, 2016.
6. C. Yang and J. Ogden, Determining the lowest-cost hydrogen delivery mode. *Int. J. Hydrogen Energy*, 32, 268–286, 2007.
7. North American Midstream Infrastructure Through 2035 – A Secure Energy Future Report, The INGAA Foundation, Inc. technical report, 2014.
8. S. Schoebnung, Economic Analysis of Large-Scale Hydrogen Storage for Renewable Utility Applications, Sandia National Laboratories report, 2011.
9. S. Samsatli and N. J. Samsatli. A multi-objective MILP model for the design and operation of future integrated multi-vector energy networks capturing detailed spatio-temporal dependencies. *Appl. Energy*, 220, 893–920, 2018.
10. Berliner, M. D., Cogswell, D. A., Bazant, M. Z. & Braatz, R. D. Methods—PETLION: Open-source software for millisecond-scale porous electrode theory-based lithium-ion battery simulations. J. Electrochem. Soc. 168, 090504 (2021).
11. Ciez RE., Steingart D., Asymptotic Cost Analysis of Intercalation Lithium-Ion Systems for Multi-hour Duration Energy Storage, Joule, 4, 597-614 (2020).
12. Pinson, M. B. & Bazant, M. Z. Theory of SEI Formation in rechargeable batteries: Capacity fade, accelerated aging and lifetime prediction. *J. Electrochem. Soc.* 160, A243–A250, 2013.
